# DpdtC-Induced EMT Inhibition in MGC-803 Cells Was Partly through Ferritinophagy-Mediated ROS/p53 Pathway

**DOI:** 10.1155/2020/9762390

**Published:** 2020-03-12

**Authors:** Jiankang Feng, Cuiping Li, Ruifang Xu, Yongli Li, Qi Hou, Rui Feng, Senye Wang, Lei Zhang, Changzheng Li

**Affiliations:** ^1^Departement of Molecular Biology and Biochemistry, Xinxiang Medical University, Xinxiang, Henan, China 453003; ^2^Experimental Teaching Center of Biology and Basic Medical Sciences, Sanquan College of Xinxiang Medical University, Xinxiang, Henan, China 453003; ^3^Department of Histology and Embryology, Sanquan College of Xinxiang Medical University, Xinxiang, Henan, China 453003; ^4^Laboratory of Molecular Medicine, Xinxiang Medical University, Xinxiang, Henan, China 453003

## Abstract

Epithelial-mesenchymal transition (EMT) is a cellular process in which epithelial cells are partially transformed into stromal cells, which endows the polarized epithelium cells more invasive feature and contributes cancer metastasis and drug resistance. Ferritinophagy is an event of ferritin degradation in lysosomes, which contributes Fenton-mediated ROS production. In addition, some studies have shown that ROS participates in EMT process, but the effect of ROS stemmed from ferritin degradation on EMT has not been fully established. A novel iron chelator, DpdtC (2,2′-di-pyridylketone dithiocarbamate), which could induce ferritinophagy in HepG2 cell in our previous study, was used to investigate its effect on EMT in gastric cancer cells. The proliferation assay showed that DpdtC treatment resulted in growth inhibition and morphologic alteration in MGC-803 cell (IC_50_ = 3.1 ± 0.3 *μ*M), and its action involved ROS production that was due to the occurrence of ferritinophagy. More interestingly, DpdtC could also inhibit EMT, leading to the upregulation of E-cadherin and the downregulation of vimentin; however, the addition of NAC and 3-MA could attenuate (or neutralize) the action of DpdtC on ferritinophagy induction and EMT inhibition, supporting that the enhanced ferritinophagic flux contributed to the EMT inhibition. Since the degradation of ferritin may trigger the production of ROS and induce the response of p53, we next studied the role of p53 in the above two-cell events. As expected, an upregulation of p53 was observed after DpdtC insulting; however, the addition of a p53 inhibitor, PFT-*α*, could significantly attenuate the action of DpdtC on ferritinophagy induction and EMT inhibition. In addition, autophagy inhibitors or NAC could counteract the effect of DpdtC and restore the level of p53 to the control group, indicating that the upregulation of p53 was caused by ferritinophagy-mediated ROS production. In conclusion, our data demonstrated that the inhibition of EMT induced by DpdtC was realized through ferritinophagy-mediated ROS/p53 pathway, which supported that the activation of ferritinophagic flux was the main driving force in EMT inhibition in gastric cancer cells, and further strengthening the concept that NCOA4 participates in EMT process.

## 1. Introduction

Gastric cancer (GC) is the second highest mortality among cancers worldwide, and higher cases of it occur in East Asia [1, 2]. Although significant improvement in both diagnosis and treatment was achieved, the overall survival rate is still poor. Therefore, more efforts to clarify the underlying mechanisms of this deadly cancer are urgently needed. Clinically, the chemotherapy is still the main treatment for advanced GC [3]; however, attenuating the side effects and resistance of chemotherapeutic agents requires a different strategy. Iron is an essential element for cell growth, and it has been demonstrated that cancer cells have higher iron demand compared to normal cells; disturbing homeostasis of iron may achieve growth inhibition of cancer cells, thus chelation therapy was proposed in clinical practice. Dithiocarbamate is an important class of sulfur-containing compounds, showing their potent applications on disease treatment, the treatment of bacterial, fungal infections, AIDS, and cancer [4, 5]. Dithiocarbamate has strong affinity toward metal ions; however, the underlying mechanism remains obscure.

Epithelial-to-mesenchymal transition (EMT) is a cellular process that epithelial cells will undergo several biochemical alterations, such as the suppression of epithelial markers and the upregulation of mesenchymal markers, endowing the polarized epithelium cells more invasive feature [6–8]. EMT is considered as a crucial step in cancer metastasis, and transforming growth factor (TGF), the cytokine, and nuclear receptor, receptor tyrosine kinase (RTK), the Wnt, Notch, hedgehog, hippo, and pathways have all been implicated in the onset of EMT [9, 10]. In addition, it has shown that the cells underwent EMT have an ability to resist conventional treatments [10]. Therefore, to develop new diagnostic and therapeutic strategies in treatment of metastases, the details in EMT required to be revealed.

ROS are constantly generated inside cells through a serial of dedicated enzyme complexes or as by-products of redox reactions, such as mitochondrial respiration [10–12]. In addition, the iron either in lysosome due to the occurrence of ferritinophagy or in labile iron pool (LIP) catalyzes Fenton reaction, yielding extremely reactive hydroxyl radicals [13]. Furthermore, the compelling evidence reveals reactive oxygen species (ROS) engage EMT process [14], but the functions of ROS remains to be determined. Recently a series of small molecular compounds exhibit the ability in EMT reversal, different signal pathway were proposed [15–18].

p53 is one of most important transcription factor, regulating proliferation, apoptosis, cell cycle and autophagy that maintain normal cellular homeostasis, controlling cell fates [19]. However, the tumor suppressor gene p53 is the most commonly mutated gene in all human cancers [20–22], such as hepatocellular carcinoma, colorectal cancer, lymphoma, mucosal melanoma, and stomach cancer [23–27]. High invasion and metastasis is the hallmark of cancer cells, EMT is prerequisite for primary cancer metastasizing to blood, and other organs [28, 29]. p53 mutation is essential to EMT process and also plays role in EMT [19, 30]. Since mutant p53 alleles may exhibit certain unique characteristics in cancer development and progression, therefore, reactivation or degradation of mutant p53 may be another strategy in cancer therapy [31]. Wild-type p53 is maintained at a low level by continuous degradation via proteasome through E3 ubiquitin ligase [30, 32], but mut-p53 degradation is diversified [33–35].

Ferritin is a highly conserved iron storage protein which is composed of two subunits, H-ferritin and L-ferritin, and the twelve pairs of subunits binding head to foot form the 24 subunit ferritin cages [36]. Ferritin degradation results in the release of iron for either use by the cell or enhancing labile iron pool; therefore, ferritin also regulates cellular redox balance. Iron chelator can lead to ferritin degradation either through ubiquitination or autophagy; the latter requires participation of microtubule-associated protein light chain 3 (LC3) and NCOA4, termed “ferritinophagy [37]. Iron depletion by some iron chelators resulted in the occurrence of ferritinophagy has been reported [37, 38], but the correlation between EMT and ferritinophagy induced by iron chelator remains to be determined. In our previous work, we reported that activating ferritinophagic flux (NCOA4/ferritin) could inhibit EMT in DpdtpA treated CT26 cell, firstly revealing that NCOA4 also involves in EMT process. To verify if this phenomenon is restricted to CT26 cells or if it represents a more general phenomenon, the gastric cancer cell line and a novel iron chelator, DpdtC were chosen in the present study. As expected, the DpdtC as DpdtpA acted also displayed EMT inhibition. The mechanistic study further supported that ferritinophagy-mediated EMT inhibition was through by activation of ROS/p53 pathway.

## 2. Results

### 2.1. The DpdtC-Induced Growth Inhibition Involved ROS Production

**Figure 1:**
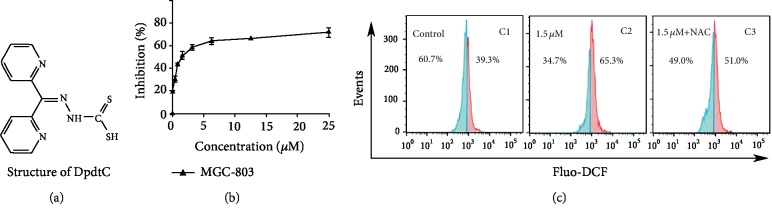
DpdtC-induced growth inhibition involved ROS generation. (a) Structure of DpdtC; (b) the effect of DpdtC on proliferation of gastric cancer cell; (c) DpdtC-induced ROS production in MGC-803 cell line: C1, control (H_2_O); C2, 1.5 *μ*M DpdtC; C3, 1.5 *μ*M DpdtC+NAC (1.5 mM). The data from MTT assay were from five measurements; and ROS assays were conducted twice.

In our previous study, 2,2′-di-pyridineketone hydrazone dithiocarbamate (DpdtC, Figure 1(a)) displayed significant antitumor activity against HepG2 cell [38]. To expand our understanding of the agent, we further studied its growth inhibitory effect on gastric cancer cells. The dose-response curve is depicted in Figure 1(b). As expected, DpdtC displayed significant growth inhibition for gastric cells (IC_50_: 3.1 ± 0.3 *μ*M for MGC-803). Furthermore, to determine whether the growth inhibition involved ROS production, the cellular ROS level was measured by flow cytometry as described previously [38]. As shown in Figure 1(c), the populations in higher fluorescence intensities significantly increased by ~30% after exposure of DpdtC to the cells for 24 h, but the addition of NAC, a ROS scavenger, significantly decreased ROS production (~14%), hinting that the antiproliferative action involved ROS production. Furthermore, the role of ROS production in the growth inhibition was further determined. As showed in [Supplementary-material supplementary-material-1], the addition of NAC (0.15 mM) could attenuate the inhibitory ability of DpdtC on proliferation of gastric cancer cells, indicating that DpdtC-induced growth inhibition was related to ROS production.

### 2.2. DpdtC-Induced ROS Was Partly due to the Occurrence of Ferritinophagy

**Figure 2:**
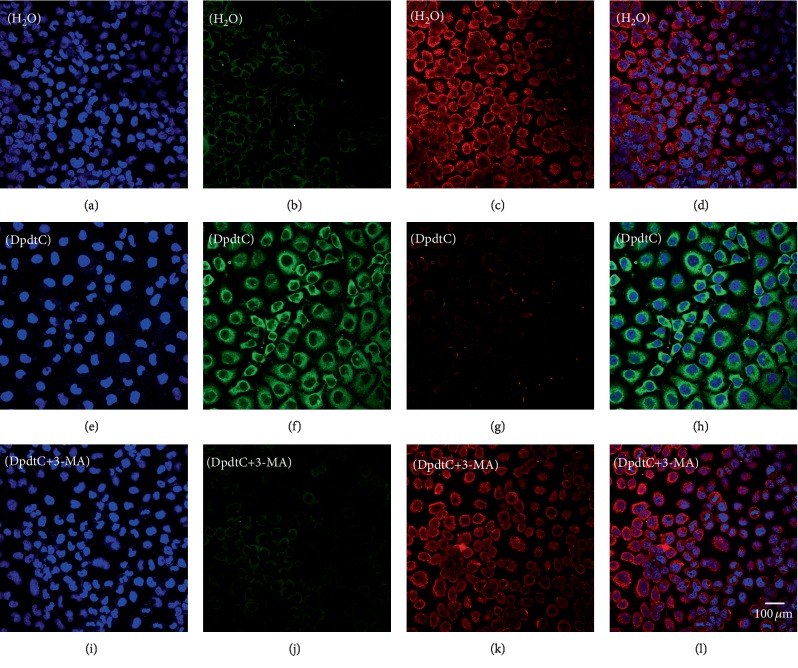
DpdtC-induced ferritin autophagy (ferritinophagy). The nuclei stained by DAPI in blue, ferritin labeled in red; NCOA4 labeled in green. (a–d) Control group: (a) nuclei in blue; (b) NCOA4 in green; (c) ferritin in red; and (d) merge of ferritin with NCOA4. (e–h) DpdtC-treated group: (e) nuclei in blue; (f) NCOA4 in green; (g) ferritin in red; and (h) merge of ferritin with NCOA4. (i–l) DpdtC combined with 3-MA group: (i) nuclei in blue; (j) NCOA4 in green; (k) ferritin in red; and (l) merge of ferritin with NCOA4. The experiments were performed thrice. Objective size: 40 × 10; scale bar: 100 *μ*m.

**Figure 3:**
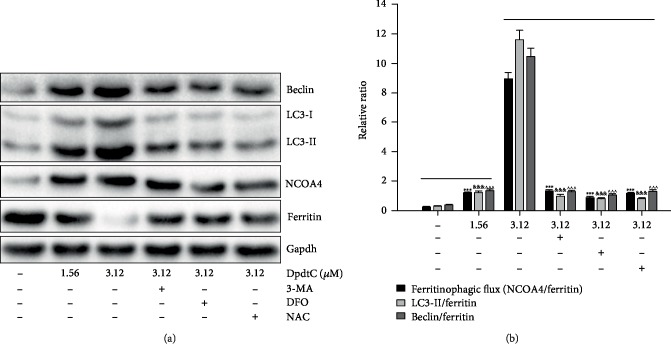
DpdtC exposure resulted in the alterations of ferritinophagy- and autophagy-related proteins. (a) Western blotting analysis of autophagic and ferritinophagic proteins; (b) the quantitative analyses of ratio of NCOA4/ferritin, LC3-II/ferritin, and beclin/ferritin at protein level from (a). The quantification analysis of indicated ratio was from two experiments. ^∗∗∗,&&&,∧∧∧^*p* < 0.01.

Previous work revealed that the DpdtC induced ferritinophagy in hepatocellular carcinoma cells [38], which led to ROS generation. DpdtC treatment in gastric cancer cells also resulted in ROS production, we hypothesized that the ROS production might be due to the occurrence of ferritinophagy. To this end, the levels of ferritin and NCOA4, a specific carrier for ferritinophagy, was evaluated via immunofluorescence technique. As shown in Figure 2, DpdtC exposure indeed resulted in upregulated NCOA4 (compare Figure 2(f) to Figure 2(b)) and downregulated ferritin (compare Figure 2(g) to Figure 2(c)), indicating that the ferritin degradation was through autophagic proteolysis. Furthermore, the addition of 3-MA, an autophagy inhibitor, could abolish the ferritinophagy, i.e., leading to a significant upregulation of ferritin (compare Figure 2(k) to Figure 2(g)). The merged photographs (Figures 2(d), 2(h), and 2(l)) clearly showed the alterations in NCOA4 and ferritin. To corroborate the occurrence of ferritinophagy, the levels of ferritin, NCOA4, and autophagy-related proteins before and after DpdtC treatment were further determined by Western blotting. As shown in Figure 3, concomitant to the decrease of ferritin, the upregulated autophagic markers (LC3-II, beclin) and NCOA4 were observed upon DpdtC treatment; however, the addition of 3-MA or DFO, as well as NAC, could markedly attenuate the levels of those upregulated proteins. Accordingly, a quantitative comparison in ratios of NCOA4/ferritin that termed “ferritinophagic flux” [39], LC3-II/ferritin and beclin/ferritin is shown in Figure 3(b), clearly DpdtC treatment resulted in activation of autophagy, i.e., increase of ferritinophagic flux and ferritin degradation. In addition, the occurrence of ferritinophagy would result in the change of cellular iron; thus, the total iron content before and after DpdtC exposure to the gastric cells was determined by atomic absorbance spectrometry. As shown in [Supplementary-material supplementary-material-1], DpdtC treatment indeed resulted in markedly decrease of iron abundance, in accordance with results from immunofluorescence and Western blotting analysis.

### 2.3. The DpdtC Induced an EMT Inhibition

**Figure 4:**
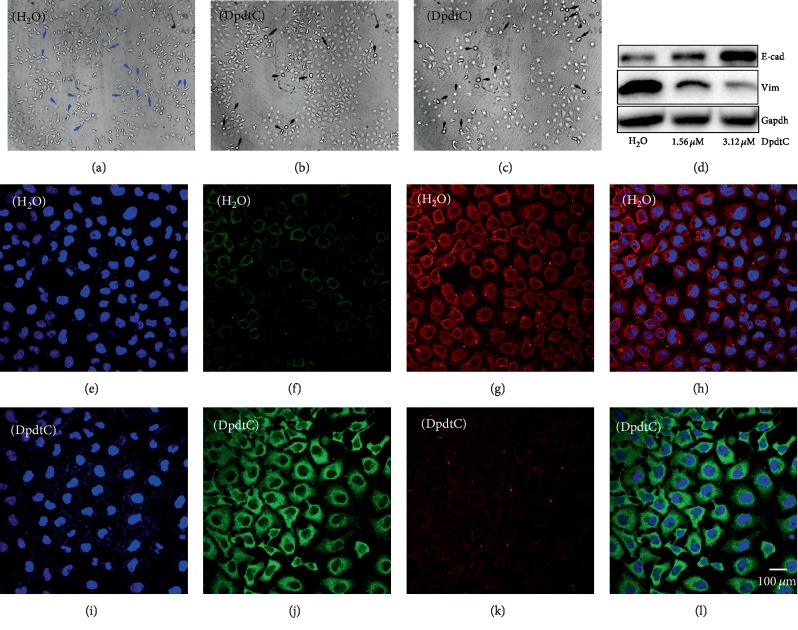
DpdtC-induced morphologic change correlated with EMT modulation. (a–c) Morphologic change upon exposure DpdtC to MGC-803 cells for 48 h. The blue arrow: spindle-shaped cells, black arrow: retracted and rounded cells; (a) H_2_O, (b) 0.31 *μ*M DpdtC, and (c) 0.62 *μ*M DpdtC; objective size: 20 × 10; scale bar: 200 *μ*m. (d) Western blotting analysis. (e–l) immunofluorescence analysis of epithelial-mesenchymal markers. (e–h) Control group (H_2_O): (e) nuclei in blue; (f) E-cadherin in green; (g) vimentin in red; and (h) merge of nuclei, E-cadherin, and vimentin in control group. (i–l) DpdtC-treated group: (i) nuclei in blue; (j) E-cadherin in green; (k) vimentin in red; and (l) merge of nuclei, E-cadherin, and vimentin in DpdtC-treated group. Objective size: 40 × 10 (fluorescence); scale bar: 100 *μ*m.

In addition to growth inhibition, DpdtC also had effect on cellular morphology of gastric cancer cells. Figures 4(a)–4(c) showed the alteration in morphology when exposure of DpdtC to the cells, which encouraged us to consider whether its action involved EMT transformation, inhibiting metastasis of cancers [40]. Thus, the molecular markers in EMT transformation were labeled by immunofluorescence technique before and after DpdtC treatment. As shown in Figure 4, the green fluorescence of E-cadherin was increased (compare Figure 4(j) to Figure 4(f)), while the red fluorescence which represented vimentin was decreased (compare Figure 4(g) to Figure 4(k)). The merged photographs (Figures 4(h) and 4(l)) clearly showed the alterations in E-cadherin and vimentin, the proportion of red fluorescence (vimentin) in Figure 4(h) was much higher than that in Figure 4(l), indicating that DpdtC could inhibit the EMT transition. Furthermore, Western blotting analysis also demonstrated that DpdtC could downregulate mesenchymal marker, vimentin, contrarily upregulate epithelial marker, E-cadherin (Figure 4(d)), in consistent with that from immunofluorescence analysis, supporting that DpdtC had inhibitory effect on EMT transformation.

### 2.4. DpdtC-Suppressed TGF-*β*1-Induced EMT through Ferritinophagy Pathway

**Figure 5:**
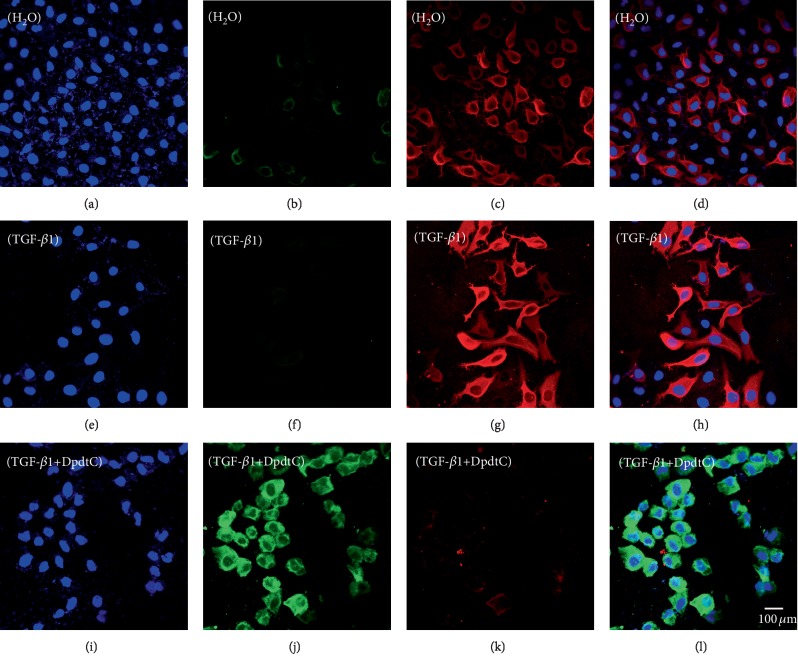
DpdtC-resisted TGF-*β*1-induced EMT. (a–d) Control: (a) nuclei stained by DAPI in blue (DMSO); (b) E-cadherin in green; (c) vimentin in red; and (d) merge of nuclei, E-cadherin, and vimentin. (e–h) TGF-*β*1 treated: (e) nuclei in blue; (f) E-cadherin in green; (g) vimentin in red; and (h) merge of nuclei, E-cadherin, and vimentin. (i–l) TGF-*β*1 treatment plus DpdtC: (i) nuclei stained by DAPI in blue (DMSO); (j) E-cadherin in green; (k) vimentin in red; and (l) merge of nuclei, E-cadherin, and vimentin. The measurements were performed thrice from different field of view. Objective size: 40 × 10; scale bar: 100 *μ*m.

To validate the ability of DpdtC in EMT modulation, a model that was undergoing EMT required to be established. Thus, the MGC-803 cells were pretreated with TGF-*β*1, the most powerful EMT inducer for 48 h, which resulted in obviously morphological alteration. As shown in Figures 5(g) and 5(h), the MGC-803 cells became more spindle-shaped, fibroblast-like cells compared to control (Figures 5(c) and 5(d)), which was considered cells undergoing the EMT [41]. Next, the undergoing EMT cells were further treated by DpdtC, as shown in Figure 5(j), DpdtC could significantly increase the level of E-cadherin and neutralize the action of TGF-*β*1 on vimentin modulation (Figure 5(k)). The alterations in E-cadherin and vimentin before and after treatment of DpdtC could be clearly observed in the merged photographs (Figures 5(h) and 5(l)), corroborating that DpdtC was able to resist TGF-*β*1-induced EMT.

**Figure 6:**
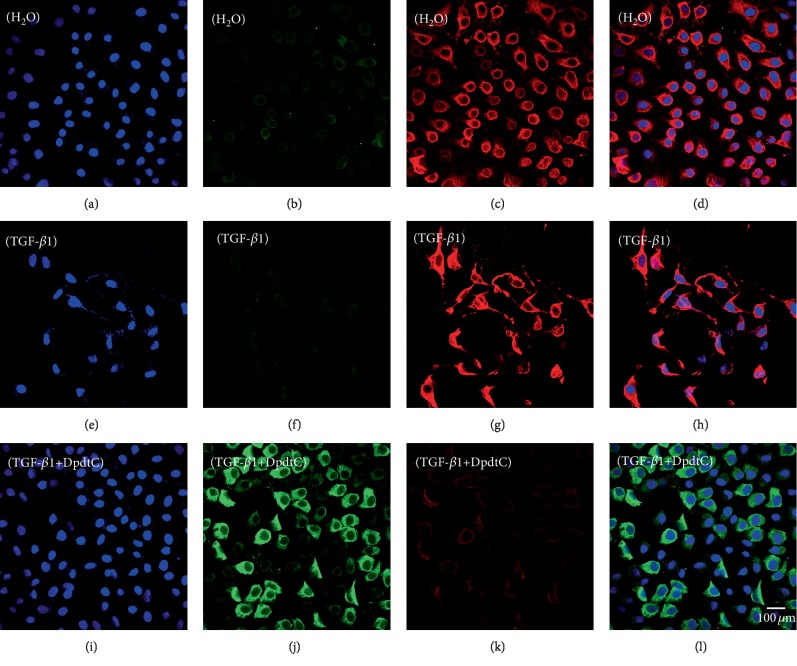
DpdtC-induced ferritinophagy in the presence of TGF-*β*1. The nuclei stained by DAPI in blue, NCOA4 in green, and ferritin in red. (a–d) Control: (a) nuclei in blue; (b) NCOA4 in green; (c) ferritin in red; and (d) merge of nuclei, ferritin, and NCOA4. (e–h) TGF-*β*1 treatment only: TGF-*β*1 treatment: (e) nuclei in blue; (f) NCOA4 in green; (g) ferritin in red; (h) merge of nuclei, ferritin, and NCOA4; (i–l) TGF-*β*1 combined with DpdtC: (i) nuclei in blue; (j) NCOA4 in green; (k) ferritin in red; and (l) merge of nuclei, ferritin, and NCOA4. The measurements were performed thrice from different field of view. Objective size: 40 × 10; scale bar: 100 *μ*m.

Since DpdtC could resist the action of TGF-*β*1 on EMT modulation, was there an occurrence of ferritinophagy in above experimental condition? Thus, the levels of cellular NCOA4 and ferritin in the presence of TGF-*β*1 were further evaluated by immunofluorescence. As shown in Figure 6, TGF-*β*1 slightly increased the level of ferritin compared to control (Figures 6(g) and 6(c)); however, the addition of DpdtC significantly attenuated the action of TGF-*β*1 on ferritin modulation (Figure 6(k)), leading to marked increase of NCOA4, indicating that DpdtC still induced ferritinophagy in gastric cancer cells even the cells were undergoing EMT. The knockdown of NCOA4 by interfering RNA resulted in increase in vimentin, slug, and snail and also neutralized the action of DpdtC on those genes modulation ([Supplementary-material supplementary-material-1]), indicating that NCOA4 indeed modulated EMT progress, in accordance with previous observation in CT26 cells [39].

**Figure 7:**
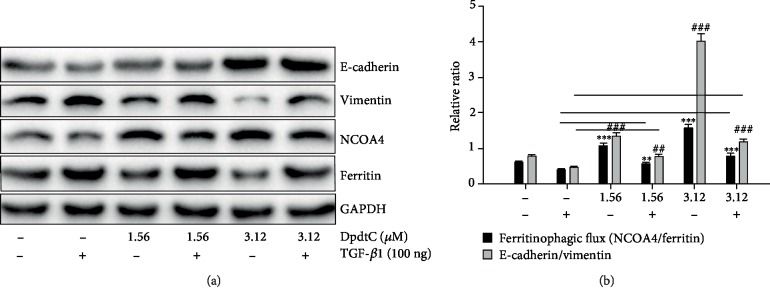
Ferritinophagic flux played an important role in EMT process. (a) The alterations in ferritinophagy- and EMT-related proteins when either TGF-*β*1 or combined with DpdtC treatment; (b) quantitative analysis of the ferritinophagic flux in the indicated conditions. The quantification analysis of related proteins was from three experiments (^∗∗,##^*p* < 0.05; ^∗∗∗,###^*p* < 0.01).

To strengthen the relationship between EMT inhibition and ferritinophagy induction, a correlation analysis between ferritinophagic flux (NCOA4/ferritinophagy) and levels of epithelial-mesenchymal markers were performed. As shown in Figure 7, TGF-*β*1 boosted EMT (upregulated vimentin and downregulated E-cadherin) through lowering ferritinophagic flux, contrarily EMT inhibition induced by DpdtC was through elevating ferritinophagic flux (Figure 7(b)). Those indicated that the status of EMT may depend on strength of ferritinophagic flux.

### 2.5. DpdtC-Suppressed EMT Was p53 Dependent

**Figure 8:**
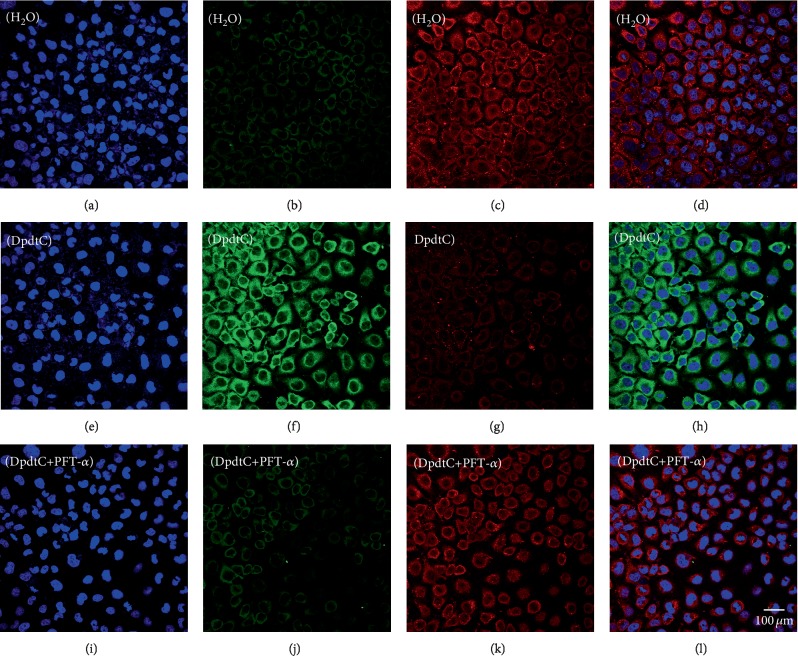
DpdtC-induced EMT modulation was p53 dependent. (a–d) Control (H_2_O): (a) nuclei in blue; (b) E-cadherin in green; (c) vimentin in red; and (d) merge of nuclei, E-cadherin, and vimentin in control group. (e–h): DpdtC-treated group: (e) nuclei in blue; (f) E-cadherin in green; (g) vimentin in red; and (h) merge of nuclei, E-cadherin, and vimentin in DpdtC-treated group. (i–l): DpdtC combined with PFT-*α*-treated group: (i) nuclei in blue; (j) E-cadherin in green; (k) vimentin in red; and (l) merge of nuclei, E-cadherin, and vimentin in DpdtC combined with PFT-*α*-treated group. The measurements were performed thrice from different field of view. Objective size: 40 × 10 (fluorescence); scale bar: 100 *μ*m.

Since mutant p53 exhibits certain unique characteristics in cancer development, p53 mutation is essential to EMT process [19, 30]. The observation that DpdtC could inhibit EMT promoted us to consider that the action of DpdtC may involve the alteration of p53 in gastric cancer cells. To this end, the PFT-*α*, a p53 inhibitor, was used to determine whether p53 involved the EMT inhibition. As shown in Figure 8, DpdtC treatment resulted in an upregulated E-cadherin (compare Figure 8(b) with Figure 8(f)) and a downregulated vimentin (compare Figure 8(c) with Figure 8(g)); however, the addition of PFT-*α* attenuated the upregulation of E-cadherin induced by DpdtC (Figure 8(f) and Figure 8(j)), accordingly enhancing vimentin expression (Figure 8(g) and Figure 8(k)), indicating that p53 indeed involved the action of DpdtC on EMT modulation. The alterations in E-cadherin and vimentin at different conditions could be clearly observed in the merged photographs (Figures 8(d), 8(h), and 5(l)). The additional evidence from Western blotting analysis supported the above conclusion. [Supplementary-material supplementary-material-1] showed that DpdtC significantly upregulated p53 and E-cadherin expression and downregulated EMT-related genes expression, such as snail, slug, and vimentin; however, a p53 inhibitor, PFT-*α*, markedly attenuated action of DtdtC on those genes modulation, which was in accordance with result from immunofluorescence.

### 2.6. DpdtC Triggered Ferritinophagy Was p53 Dependent

**Figure 9:**
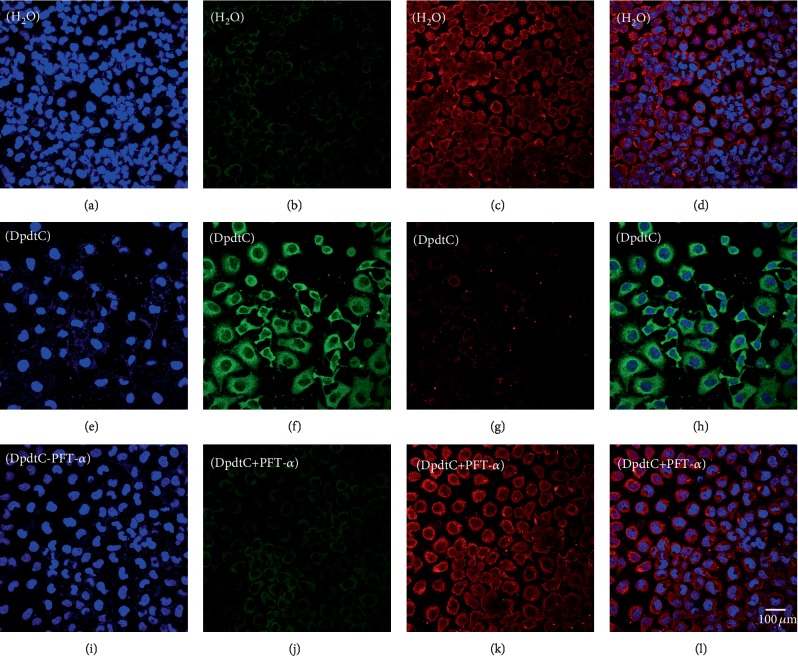
DpdtC-induced ferritinophagy involved p53. The nuclei stained by DAPI in blue, ferritin labeled in red, and NCOA4 labeled in green. (a–d) Control group: (a) nuclei in blue; (b) NCOA4 in green; (c) ferritin in red; and (d) merge of ferritin with NCOA4. (e–h) DpdtC-treated group: (e) nuclei in blue; (f) NCOA4 in green; (g) ferritin in red; and (h) merge of ferritin with NCOA4. (i–l) DpdtC combined with PFT-*α* group: (i) nuclei in blue; (j) NCOA4 in green; (k) ferritin in red; and (l) merge of ferritin with NCOA4. The measurements were performed thrice from different field of view. Scale bar: 100 *μ*m.

Since DpdtC-induced EMT inhibition was p53 dependent, the ferritinophagy induction might be in similar manner. Thus cell were treated with DpdtC alone or combined with PFT-*α* for 24 h, then the level of ferritinophagy-related protein was investigated by immunofluorescence technique. As shown in Figure 9, the addition of PFT-*α* indeed reversed the action of DpdtC in ferritinophagy induction (Figure 9 H and 9 L), indicating that the action of DpdtC involved p53 response. To seek additional evidence, the expressions of EMT and ferritinophagy-related proteins were detected further by Western blotting. As shown in [Supplementary-material supplementary-material-1], DpdtC could upregulate NCOA4 and downregulate ferritin; however, this action could significantly attenuate PFT-*α*, indicating that the upregulation of NCOA4 correlated with the upregulation of p53.

### 2.7. Ferritinophagy-Mediated ROS Resulted in the Upregulation of p53 and Growth Inhibition

**Figure 10:**
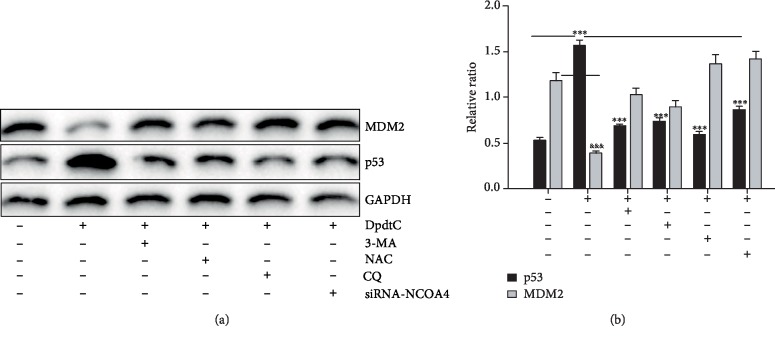
p53 responded to ROS production induced by DpdtC. (a) The upregulation of p53 could be attenuated by the addition of autophagy inhibitor, ROS scavenger, and the downregulation of NCOA4; (b) quantification of the changes of p53 and MDM2 from (a). The experiments were performed twice. ^∗∗∗,&&&^*p* < 0.01.

As showed in Figure 3, DpdtC could induce autophagy, which led to the upregulation of beclin and LC3-II, with a concomitant accumulation of ferritinophagic flux (the ratio of NCOA4/ferritin increased). The occurrence of ferritinophagy undoubtedly generated excessive ROS due to ferric iron release triggering Fenton reaction, accordingly leading to p53 response to initiate either protect the cell from death or death program. [Supplementary-material supplementary-material-1] showed that DpdtC-induced ROS production could be attenuated by the addition of a p53 inhibitor, PFT-*α*, indicating that p53 responded ferritinophagy-mediated ROS production. Additional evidence was from Figure 10; the DpdtC-induced upregulation of p53 could be attenuated by autophagy inhibitor, 3-MA, and chloroquine (conversely the level of MDM2 was enhanced), indicating that the occurrence of autophagy responded the rising of p53. Both knockdown of NCOA4 by interfering RNA and addition of NAC all neutralized upregulation of p53 induced by DpdtC, further supporting that ferritinophagy-mediated ROS triggered p53 activation. Similarly, situation occurred in growth inhibition induced by DpdtC ([Supplementary-material supplementary-material-1]). Taken together, those supported that ROS (originated from ferritinophagy)/p53 involved EMT inhibition induced by DpdtC.

## 3. Discussion

Tumor metastasis undergoes epithelial-mesenchymal transition (EMT), which endows cells with invasive ability [40]. Therefore, EMT inhibition may be helpful for tumor therapy. A number of transcription factors, such as ZEB 1 and ZEB 2, snail, slug, and twist, regulate EMT [41]. Furthermore, the growth factors and cytokines, ECM components, hypoxia, ROS, and other factors also observed to be involved in EMT process [42]; therefore, the regulation of EMT is diversified and remains to be elucidated. In previous study, we observed that DpdtpA could both inhibit proliferation and EMT in CT26 cells. DpdtC, an analogue of DpdtpA, may have similar function in gastric cancer line. Thus, the current study was focused on the alteration of the MGC-803 cells upon DpdtC treatment. Similar to DpdtpA, DpdtC displayed a significant growth inhibition that was attenuated by the addition of NAC, indicating that the induced growth inhibition was ROS dependent (Figures 1 and [Supplementary-material supplementary-material-1]); similar situations were observed in others studies [39, 43]. ROS production may stem from lysosomes, mitochondria, and imbalance between cellular oxidants and antioxidants; thus, the possible origin of ROS generation in DpdtC-induced growth inhibition needed to be determined. Generally, the main types of ROS from mitochondria are superoxide and peroxide [44], while the hydroxyl radical is mainly due to Fenton reaction occurred in lysosomes or labile iron pool. In our study, DpdtC caused marked decrease of ferritin, accompanied by increasing of NCOA4 and autophagy-related proteins, revealing that the ROS production partly stemmed from the occurrence of ferritinophagy (Figures 2, 3, and [Supplementary-material supplementary-material-1]), in accordance with observation in HepG2 cells reported previously [38]. The morphologic alteration of MGC-803 induced by DpdtC happened in similar way in CT26 cells caused by DpdtpA, which implied that the action of DpdtC might involve EMT regulation. The immunofluorescence and Western blotting analysis confirmed our speculation because of the alteration in epithelial-mesenchymal markers after DpdtC treatment (Figure 4), in accordance with others observation [45]. To corroborate the ability of DpdtC in induction, the EMT model that induced by TGF-*β*1 needed to be established for this method has been widely accepted in molecular biology [46]. It was interesting that DpdtC abolished the action of TGF-*β*1 on EMT induction in the present study (Figure 5), demonstrating that DpdtC was able to inhibit EMT, which was in accordance with the action of the iron chelator reported previously [47]. Next, we questioned whether DpdtC could still trigger ferritinophagy in the presence of TGF-*β*1. Figure 6 showed that an upregulation of NCOA4 and a downregulation of ferritin occurred as in absence of TGF-*β*1, supporting that ferritinophagy induced by DpdtC was independent of EMT process. DpdtC induced the upregulation of NCOA4, leading to EMT inhibition, and the knockdown of NCOA4 by interfering RNA resulted in the upregulation of mesenchyma-related proteins (vimentin, slug, and snail) (Figure 7 and [Supplementary-material supplementary-material-1]), indicating that NCOA4 participated in EMT. We speculated that there could be a correlation between ferritinophagy and EMT inhibition; thus, “ferritinophagic flux” (defined as ratio of NOCA4/ferritin) was used to correlate with the change in EMT. As shown in Figure 7, DpdtC and TGF-*β*1 treatment resulted in adverse variation in ferritinophagic flux, the former increase, and the later decrease, so we may also describe that the EMT status is related to ferritinophagic flux, i.e., ferritinophagic flux was a dominating force in determination of EMT state in gastric cancer cells. This also provided additional evidence that NCOA4 played role in EMT modulation. Normally, NCOA4 acts as a transcriptional coactivator of several nuclear receptors, and its additional function was recently identified as an inhibitor in the activation of DNA replication origins [48]; the downregulation of snail, slug, and vimentin in DpdtC-treated cells might be due to the upregulation of NCOA4. In addition, it has been shown that ROS involved TGF-*β*1-induced EMT, and the decrease of ferritin heavy chain (FHC) contributed to EMT transition [49–51]. However, the ferritin degradation induced by DpdtC achieved EMT inhibition in our study; the difference might be attributed to a difference in manner of ferritin downregulation achieved; the knockdown of ferritin by sh-RNA was at translation level, while induced ferritinophagy by DpdtC at posttranslation modification. On the other hand, the cell specific was also a crucial factor to be considered. Importantly, DpdtC treatment resulted in the downregulation of snail, an EMT transcription factor, implying EMT inhibition achieved was complex for DpdtC. The ferritin degradation led to cellular ROS production on account of the ferric iron liberated from digested ferritin would reduce by the endosomal ferrireductase Steap3 in the acidified lysosome [52], the resulting ferrous ion triggered Fenton reaction. As a stress responder, p53 may respond to the alteration in redox environment; therefore, the p53 response was further determined. Cell growth and proliferation require checkpoint controls, while p53 as a major checkpoint protein in mammalian cells is normally kept at a low level as a result of a negative feedback regulation between p53 and Mdm2 [53, 54]. Our data showed that DpdtC-induced ferritinophagy and EMT inhibition along with the upregulation of p53, and inhibition of p53 could significantly attenuate the effects of DpdtC (Figures 8 and 9), indicating that p53 indeed responded the stress (ROS insulting). It was reported that p53 was regulated by metal ions, and pyrrolidine dithiocarbamate (PDTC) downregulated p53 due to increasing intracellular level of copper [55]. As an analogue of PDTC, but DpdtC has distinct effect on p53 regulation, the difference in p53 regulation may be strictly dependent on the cell type for there were differences in the status of p53 mutation of the cell line used and in the resulting redox environment upon treatment of the agents [56, 57]. Indeed, we also observed that the DpdtbA (a dithiocarbamate derivative) inactivated p53 in esophageal cancer cell lines as PDTC did [58]. In addition, whether the EMT inhibition induced by DpdtC (including its analogues) is related to the degradation of ferritin, and the state of p53 determines whether it occurs or not, which needs further study.

Taken all together, DpdtC induced the growth inhibition in gastric cancer cells that was ROS dependent, which mechanistically was due to the occurrence of ferritinophagy that contributed to ROS production. In addition, DpdtC was also able to inhibit EMT; the correlation analysis further demonstrated that the ferritinophagic flux was a dominating driving force during EMT development, decrease of the ferritinophagic flux resulted in EMT enhancement, while increase of ferritinophagic flux favored to inhibit EMT (reversing EMT). In addition, the p53 also played role in DpdtC-induced EMT inhibition and ferritinophagy induction; the downregulation of p53 would significantly attenuate (or abolish) the action of DpdtC. However, the correlation between ferritinophagic flux and EMT in other cell lines *in vivo* and *in vitro* requires more studies in the future, because the diversity of iron chelators in structure and cell specific influences the effect of iron chelator.

## 4. Materials and Methods

### 4.1. Materials

All chemicals used were analytical reagents (AR) grade. 3-(4,5-Dimethylthiazol-2-yl)-2,5-diphenyltetrazolium bromide (MTT), pifithrin-*α* (PFT-*α*), 3-methyladenine (3-MA), chloroquine (CQ), dichlorofluorescein (H_2_DCF-DA), desferoxamine (DFO), 4′,6-diamidino-2-phenylindole (DAPI), Roswell Park Memorial Institute (RPMI)-1640, and other chemicals were purchased from Sigma-Aldrich. Antibodies of vimentin (60330-1-lg, 10366-1-AP), NCOA4 (E11-17114C), LC3 (14600-1-AP), beclin (66665-1-Ig), and gadph (E12-052) for Western blotting were obtained from Proteintech Group Inc (Wuhan, China). Antibodies of E-cadherin (3195), ferritin (H chain, 3998S), and secondary antibodies (fluorescence labeled for immunofluorescence, 8890S, 4412S) were purchased from Cell Signaling Technology (USA). Ferritin antibody (SC-376594) for immunofluorescence was obtained from Santa Cruz Biotechnology (Santa Cruz, USA). NCOA4 antibody (HPA0512) for immunofluorescence was purchased from Atlas Antibody (Sweden). Secondary antibodies for Western blotting were obtained from EarthOx, LLC (San Francisco, USA).

### 4.2. Cytotoxicity Assay (MTT Assay)

The stock solution of DpdtC (10 mM) was prepared in water and diluted to the required concentration with culture medium when used. The MTT assay was based on reported previously [39]. Briefly, MGC-803 cells were cultured in RPMI 1640 medium supplemented with 10% fetal calf serum (FCS) and antibiotics. The cells in exponential phase were collected and seeded equivalently into a 96-well plate; next, the varied concentration of DpdtC (or other agents) was added after the cells adhered. Following 48 h incubation at 37°C in a humidified atmosphere of 5% CO_2_, 10 *μ*l MTT solution (5 mg/ml) was added and further incubated for 4 h; next, 100 *μ*l DMSO was added in each well to dissolve the formazan crystals after removing cell culture. The measurement of absorbance of the solution was performed on a microplate reader (MK3, Thermo Scientific) at 570 nm. Percent growth inhibition was defined as percent absorbance inhibition within appropriate absorbance in each cell line. The same assay was performed in triplet.

### 4.3. Flow Cytometric Analysis of Cellular ROS

MGC-803 cells were seeded into a 6-well plate and treated as described in the section of cytotoxicity assay. The cells were treated with different concentrations of the agent (1.56 and 3.12 *μ*M DpdtC) for 24 h. Then, the cell culture was removed, following PBS washing and trypsin digestion; finally, the cells were resuspended in H_2_DCF-DA containing serum-free culture medium and incubated for 30 min. Next, after removing the H_2_DCF-DA-contained medium by centrifugation and washing with PBS, the intracellular ROS assay was performed on a flow cytometer (Becton-Dickinson, USA).

### 4.4. Immunofluorescence Analysis

The immunofluorescence analysis was performed as described previously [39]. Briefly, MGC-803 cells were first cultured in a 6-well plate with cover glass overnight. The cells were treated by DpdtC for 24 h, fixed with 4% paraformaldehyde in PBS for 15 min at 37°C, and then permeabilized with 0.2% triton-X-100 in PBS for 10 min. After blocking with 1% BSA in PBS for 30 min, the cells on the cover glass were incubated with either ferritin (H chain, Santa Cruz Biotechnology), or combined with LC3 (or NCOA4 (Altas Antibodies)), or vimentin combined with E-cadherin (Cell Signaling Technology) primary antibody at 4°C for overnight. Next, removing the primary antibodies and washing with PBS, the cover glasses were further incubated with fluorescence-labeled secondary antibody for 3 h at room temperature. After removing the secondary antibody, the cells on the cover glass were further counterstained with DAPI. Finally, a confocal laser scanning microscope (Nikon eclipse Ts2, Japan) was used to visualize the cells, and the representative cells were selected and photographed.

### 4.5. Western Blotting Analysis

The Western blotting was conducted as described previously [39]; briefly, 1 × 10^7^ MGC-803 cells that treated with or without DpdtC were scraped in lysis buffer (50 mM Tris-HCl, pH 8.0, 150 mM NaCl, 1.0% NP-40, 10% glycerol, and protease inhibitors) on ice for 30 min, and the clear supernatant was stored at −80°C after centrifugation at 14,000 × g. Protein concentration was determined using a colorimetric Bio-Rad DC protein assay as recommended by the company (Life Science Research, USA). Proteins (30 *μ*g) were separated on a 13–15% sodium dodecyl sulfate-polyacrylamide gel at 200 V for 3 h. Next, the separated proteins on the gel were subsequently transferred onto a PVDF membrane at 60 V for 2 h, followed by washing with Tris-buffered saline (TBS), and the membrane was then blocked for 2 h in TBS containing 0.1% Tween-20 and 5% nonfat skimmed milk. The membrane was further incubated at 4°C overnight with the appropriate primary antibody and with the appropriate HRP-conjugated secondary antibody (1 : 2,000 in TBST) for 1 h at room temperature after removing primary antibody and washing. Finally, the membrane was washed with TBST, and the protein bands were detected using a super sensitive ECL solution (Boster Biological Technology Co. Ltd.) and visualized using a Syngene G: BOX imager (Cambridge, United Kingdom). Quantifications of protein bands intensities and fluorescence intensity were performed using ImageJ software.

### 4.6. Statistical Analysis

Results are presented as the mean ± SEM. Comparisons between two groups were carried out using the two-tailed Student's *t*-test. Comparisons between multiple groups were performed by one-way ANOVA with Dunnett post hoc correction. A *p* value <0.05 was considered statistically significant.

## Data Availability

The data used to support the findings of this study are included within the article

## Supplementary Materials

Supplementary Materials In the supplementary materials, we provided additional information that included the effects of ROS induced by DpdtC on cell growth, lower abundance of iron due to the occurrence of ferritinophagy, the knockdown of NCOA4 by small-interfering RNA resulted in enhancing EMT, and p53 involved the EMT inhibition and ferritinophagy induction, finally the occurrence of ferritinophagy contributed to ROS to support the conclusion that DpdtC-induced EMT inhibition was through ferritinophagy-mediated ROS/p53 pathway.
