# Supplementary material for: DpdtC-Induced EMT Inhibition in MGC-803 Cells Was Partly through Ferritinophagy-Mediated ROS/p53 Pathway
Source: Oxid Med Cell Longev. 2020 Mar 12;2020:9762390. doi: 10.1155/2020/9762390 (PMC7091554; doi:10.1155/2020/9762390)
Supplement: Supplementary Materials — In the supplementary materials, we provided additional information that included the effects of ROS induced by DpdtC on cell growth, lower abundance of iron due to the occurrence of ferritinophagy, the knockdown of NCOA4 by small-interfering RNA resulted in enhancing EMT, and p53 involved the EMT inhibition and ferritinophagy induction, finally the occurrence of ferritinophagy contributed to ROS to support the conclusion that DpdtC-induced EMT inhibition was through ferritinophagy-mediated ROS/p53 pathway. [file 9762390.f1.docx]

**Supplementary Materials**

**DpdtC induced growth inhibition was ROS dependent**

Since DpdtC induced ROS production, which might contribute to the growth inhibition. To this end, a ROS scavenger, NAC was used to determine the role of ROS in proliferation. As shown in Figure S1, the addition of NAC could significantly attenuate the action of DpdtC in growth inhibition induced by DpdtC (p<0.05), indicating that DpdtC induced growth inhibition was ROS dependent. Similarly this situation occurred by addition of 3-MA and p53 inhibitor, indicating that the growth inhibition involved autophagy induction, and was p53 dependent.


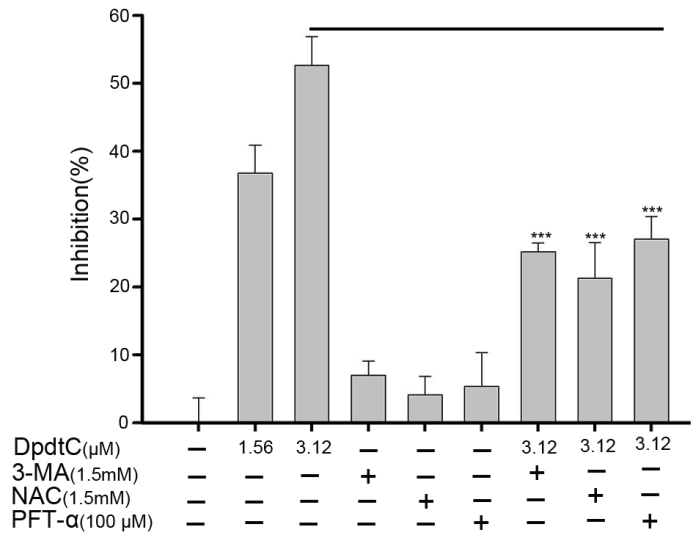


**Figure S1**: The effect of NAC, 3-MA and PFT- α on growth inhibition induced by DpdtC in MGC-803 cell. The procedure was similar to that in MTT assay. The experimental condition was as indicated. (***, p<0.05; one-way ANOVA )

**DpdtC induced decrease of total iron abundance was parallel to occurrence of ferritinophagy**

The total iron content of MGC-803 was determined by atomic absorption spectroscopy (AAS) (Persee, Beijing, China, model TAS-900) based on a protocol reported previously [1]. Briefly, the cultured cells were washed three times with a HEPES-buffered solution (154 mmol/L NaCl, 10 mM HEPES, pH 7.4) after collection through centrifugation, then the cell pellet was lysed by addition of HNO_3_ with heating, and the iron content was determined by AAS. A cell-free sample was prepared the same way and used as a blank.


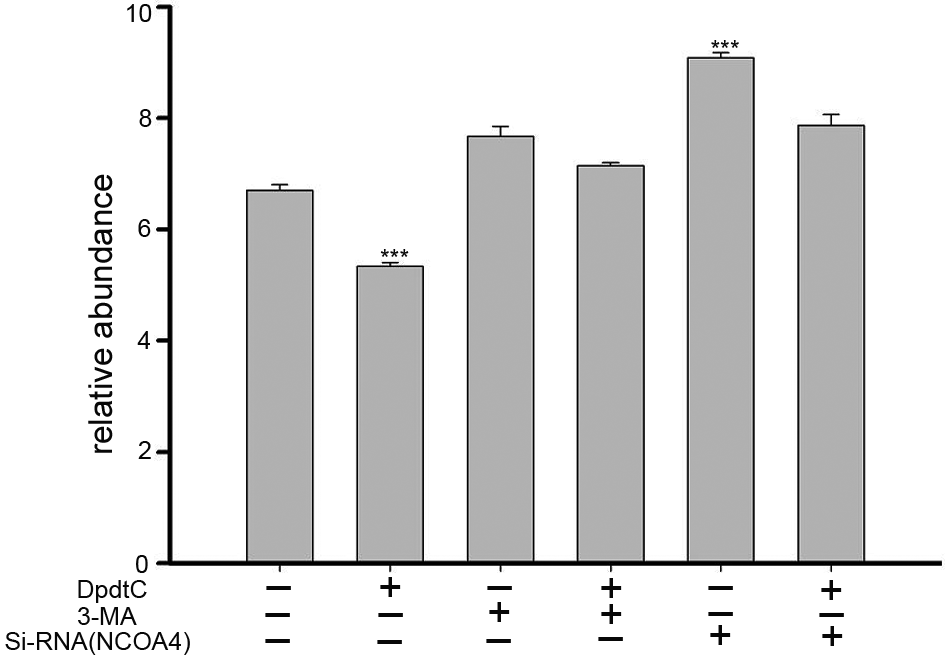


**Figure S2**: alteration of iron abundance when treatment with DpdtC or combination with 3-MA. (***, p<0.01).

**Attenuating ferritinophagic flux by knocking down of NCOA4 could promote EMT**

To test the hypothesis that NCOA4 involved in EMT, the small-interfering RNA (Si-RNA) to knockdown NCOA4. Briefly, after removing culture and washing with PBS, MGC-803 cells (1×10^6^) were transfected with 100 pmol of siRNA using Lipofectamine™ Stem Transfection Reagent (Invitrogen, USA) for 12 h as the manufacturer’s recommended protocol. Next the transfected cells were treated by DpdtC with complete medium for 24 h. The Western blotting analysis is shown in Figure S3, downregulation of NCOA4 clearly resulted in upregulation of vimentin, slug and snail, leading to mesenchymal transformation, accompanied by attenuation of ferritinophagic flux. However, the addition of DpdtC significantly attenuated the effect of small-interfering RNA on mesenchyme-related gene expression, indicating that DpdtC induced EMT inhibition indeed involved in ferritinophagy.

**
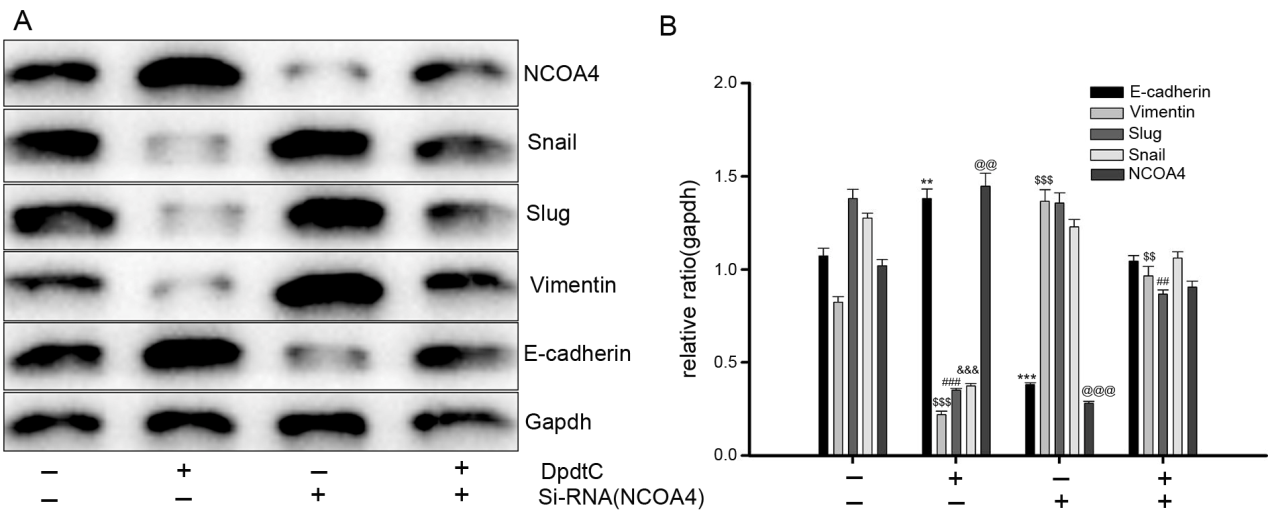
**

Figure S3: the effect of NCOA4 on EMT transformation. (A) Western blotting analysis of EMT related proteins; (B) quantification analyses of E-cadherin, vimentin, slug, snail and NCOA4. The experiments were performed thrice (^**,$$,@@,##^p<0.05 and ^***,$$$,@@@,###,&&&^p<0.01).

**DpdtC inhibited EMT was p53 dependent**

Since DpdtC treatment resulted in EMT inhibition and ROS production, as a stress responder, p53 might respond the two events. Thus a p53 inhibitor, PFT-α was used to determine the role of p53 in EMT. As shown in Figure S4, downregulation of p53 resulted in significant upregulation of slug, and slight increase of vimentin and snail compared to control. However the addition of DpdtC could attenuate the action of PFT-α on mesenchyme-related gene expression, indicating that p53 involved in EMT induced by DpdtC.


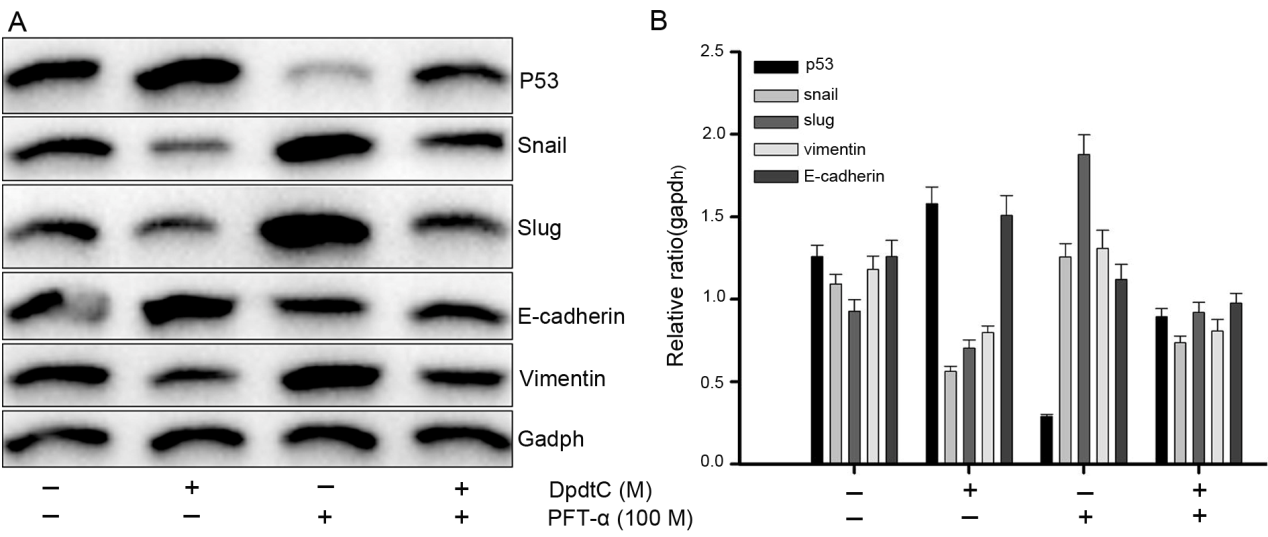


Figure S4: p53 involved EMT inhibition induced by DpdtC, the condition was indicated in the figure. (A) Western blotting analysis of alteration of EMT related proteins; (B) quantification analyses of E-cadherin, vimentin, slug, snail and p53. The experiments were performed twice.

**DpdtC induced ferritinophagy was involved in p53**

Since DpdtC induced EMT inhibition was p53 dependent, while EMT inhibition correlated with ferritinophagy, the ferritinophagy would also involve p53. To this end, the p53 inhibitor, the effect of PFT-α on ferritinophagy-related gene was evaluated. As shown in Figure S5, PFT-α downregulated NCOA4, accordingly upregulated ferritin, those could be attenuated by addition of DpdtC.


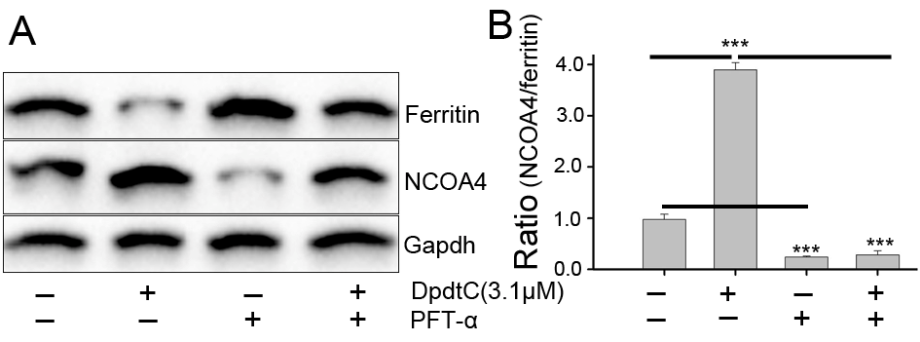


Figure S5: p53 downregulation resulted in enhancement of ferritinophagic flux. (A) The alterations in ferritinophagy-related markers when the MGC-803 cells treated by DpdtC in the absence or presence of PFT-α; (B) quantification analysis based on (A). The condition was indicated in the figure. The experiments were performed twice. (^***^P<0.01).

**ROS production induced by DpdtC was responsible for the upregulation of p53**

DpdtC induced occurrence of ferritinophagy, accordingly triggering ROS production due to Fenton reaction, consequently resulting in p53 response. To test the hypothesis, the ROS production at different conditions was assayed. As shown in Figure S6, massive ROS production (~40% increase) in DpdtC treatment compared to the control (Figure S6B), was significantly attenuated by addition of NAC (Figure S6E), supporting that the action of DpdtC involved ROS. Similarly the ROS productions were decreased by additions of 3-MA, chloroquine and si-RNA(NCOA4), respectively (Figure , indicating that the ROS may stemmed from occurrence of ferritinophagy. The addition of DFO could weak the ROS production due to its ability in ROS chelation. Furthermore p53 inhibitor also attenuated the ROS production, indicating that there was a communication between ROS and p53.


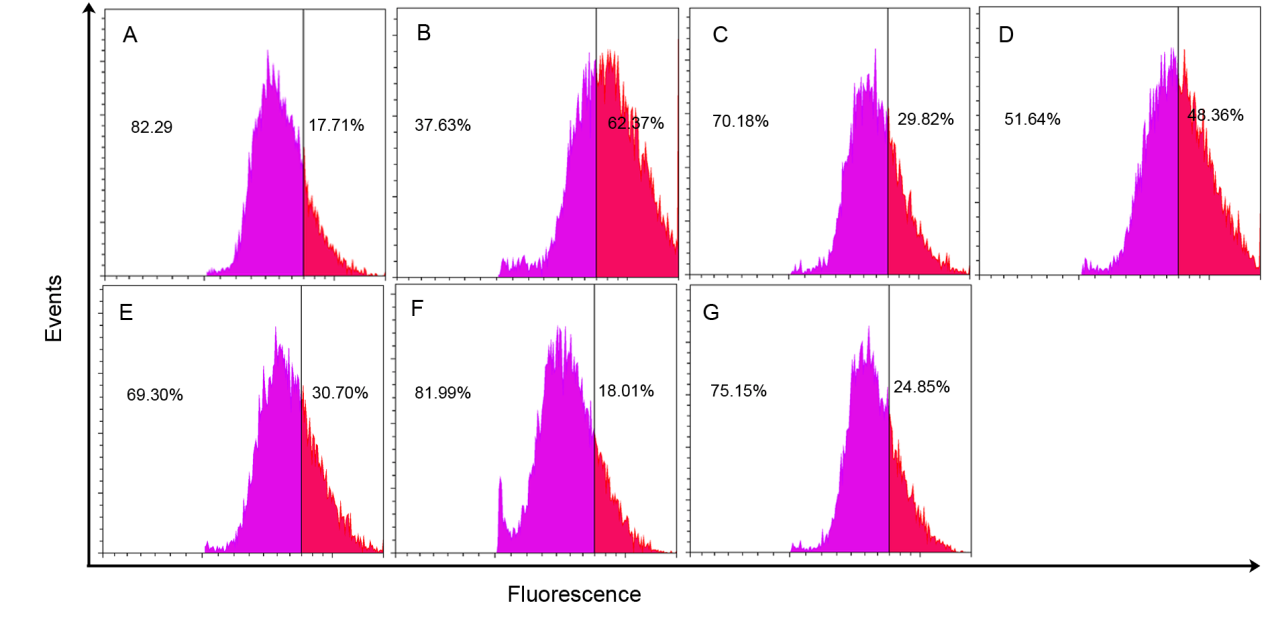


Figure S6: Autophagy inhibitor, ROS scavenger and p53 inhibitor significantly attenuated the ability of DpdtC in ROS production. (A) control; (B) DpdtC; (C) 3-MA plus DpdtC; (D) chloroquine plus DpdtC; (E) si-RNA(NCOA4) plus DpdtC; (F) NAC plus DpdtC; (G) PFT-α plus DpdtC.

**Reference**

1. Jan Riemer, Hans Hermann Hoepken, Hania Czerwinsk, Stephen R. Robinson, and Ralf Dringen, “Colorimetric ferrozine-based assay for the quantitation of iron in cultured cells,” Analytical Biochemistry 331 (2004), 370–375. µ
